# A Mathematical Model of Sentimental Dynamics Accounting for Marital Dissolution

**DOI:** 10.1371/journal.pone.0009881

**Published:** 2010-03-31

**Authors:** José-Manuel Rey

**Affiliations:** Departamento de Análisis Económico, Universidad Complutense, Madrid, Spain; RAND Corporation, United States of America

## Abstract

**Background:**

Marital dissolution is ubiquitous in western societies. It poses major scientific and sociological problems both in theoretical and therapeutic terms. Scholars and therapists agree on the existence of a sort of *second law of thermodynamics for sentimental relationships*. Effort is required to sustain them. Love is not enough.

**Methodology/Principal Findings:**

Building on a simple version of the second law we use optimal control theory as a novel approach to model sentimental dynamics. Our analysis is consistent with sociological data. We show that, when both partners have similar emotional attributes, there is an optimal effort policy yielding a durable happy union. This policy is prey to structural destabilization resulting from a combination of two factors: there is an effort gap because the optimal policy always entails discomfort and there is a tendency to lower effort to non-sustaining levels due to the instability of the dynamics.

**Conclusions/Significance:**

These mathematical facts implied by the model unveil an underlying mechanism that may explain couple disruption in real scenarios. Within this framework the apparent paradox that a union consistently planned to last forever will probably break up is explained as a mechanistic consequence of the second law.

## Introduction

Sentimental relationships of a romantic nature are typically considered a fundamental component of a balanced happy life in western societies [1]. When people are asked what they believe necessary for happiness they usually give priority to ‘love’ or to ‘a close relationship’ [2], [3], [4]. It is hard to think of another aspect of human life involving so many cultural, sociological, psychological or economic issues. Whereas the initial stage of romantic relationships seems to be controlled by chemical processes (see [5] and references therein), the issue of maintaining a sentimental relationship may rather belong in the realm of rational decisions. People usually engage in long-term relationships –typically marriage– only after due consideration. Even in the prevalent western scenario of sequential monogamy, couples generally assert their intention to make their relationship last and be happy together (see data reported in section 2). But the high divorce rates massively reported across Europe and in the United States show a resounding failure in their program implementation. The phenomenon of couple disruption is considered epidemic in the US where the statistic ‘one in two couples end in divorce’ is quoted repeatedly in the media and in academic reports. The average rate in EU27 is not far below that figure and some countries in Europe show higher rates of divorce. Furthermore, data on unmarried couples tell an even worse tale of sentimental break ups (see section 2.)

There is general agreement among scholars from different fields on mainly attributing the rise in marital instability in the twentieth century to the economic forces unleashed by the change in sexual division of labour [6], [7]. However, that reason cannot account for the ongoing and pervasive marital disruption observed in the last decades [8]. Indeed, it is not understood at this juncture why so many couples end in divorce while some others do not (see [9], pg. xi). That understanding is of paramount importance since the social change induced by marital disruption deeply affects the social structure of contemporary western societies as well as the well being of their members.

The fact that, for most couples, both partners plan enduring relationships and commit to work for them, poses a contradiction with the reportedly high divorce rates. This contradiction is referred to in this article as the *failure paradox*. According to Gottman et al [9], the field of marriage research is in desperate need of (a mathematical) theory. This paper aims to alleviate the need. In particular, it offers a consistent explanation for the failure paradox.

The work by Gottman et al –collected in [9]– seems to be the only mathematical contribution to the study of couple relationships so far. They used a pair of nonlinear difference equations estimated from the short-term interaction between two partners when observed in the lab. A simple dynamical system modelling for couple interaction was first suggested by Strogatz [10]. We adopt here a different dynamical approach: the couple is taken as a unit –no inside interaction is considered– and their sentimental dynamics is rationally prescribed by their intention to be happy together forever.

In view of the ubiquity of the phenomenon of couple break-up, it seems sensible to look beyond specific flaws in relationships and search instead for an underlying basic deterministic mechanism accounting for break-ups. Building on sociological data, we propose a mathematical model based on optimal control theory accounting for the rational planning by a homogamous couple of a long term relationship. A couple is said to be homogamous when the individual partners have similar characteristics. Homogamous mating is the most common type of sentimental partnership in western societies [11], [12]. Our model actually requires a weak form of homogamy (see section 2). We describe the evolution of this form of relationship by a dynamical equation based on the *second thermodynamic law for sentimental interaction* (*second law* for the sequel), as it has been called by Gottman et al [9]. The *second law* asserts that a sentimental relationship will deteriorate unless ‘energy’ is fed into it. This generally accepted fact allows us to model sentimental relationships as a control problem, with energy in the form of effort playing the role of the control variable. Optimal control theory has been used extensively in applied sciences, e.g. in engineering or economics. Our optimal control modelling brings a novel mathematical approach to the analysis of marriage and close relationships.

Given some feasibility conditions, our analysis of the model shows that long-term successful relationships are possible and correspond to equilibrium paths of the dynamics. While it may appear obvious that long-term relationships are not possible without some effort, a remarkable finding of the model is that the level of effort which keeps a happy relationship going is *always* greater than the effort level that would be chosen optimally a priori (if only the present counted.) Relationships are viable provided that the *effort gap* between the two levels is tolerable. The main result of the mathematical analysis is that sentimental dynamics subject to the second law are intrinsically unstable. This implies that when effort is relaxed, gradual sentimental deterioration may easily occur. The analysis identifies a plausible mechanism accounting for progressive degradation leading either to rupture or to unsatisfactory sentimental lives.

The results in the paper contribute to the resolution of the failure paradox: under the second law, the optimal design of a durable happy relationship is compatible with its dynamic instability and in turn with its probable break-up. This striking finding dismantles the failure paradox, since real relationships are expected to be subject to further sources of instability and uncertainty. Also, the results may indicate how to keep a long term relationship alive and well.

In section 2 key evidences supported by sociological data are presented that will serve as a framework to test the consistency of the model findings. The issue of the failure paradox is derived here from sociological evidence. The elements of the model are introduced along with a thorough discussion of the underlying assumptions. The main predictions of the model analysis are gathered in section 3 and some of them are shown to be consistent with facts presented in section 2. Aiming at a more fluent discussion, the mathematical technicalities are relegated to an appendix.

## Methods

### Stylized Facts

Martin and Bumpass [13] used 1985 data to show that, within a span of 40 years, two out of three marriages in the US will end in separation or divorce. This proportion may not have been reached yet but the data for 2002 show that we are not far below. About 50% of people in their early forties have already divorced at least once [14]. The much publicized figure of 50% turns out to be only slightly higher than the average divorce rate (44%) in the EU27 in 2005, and in some European countries this proportion is as high as 71% [15].

The figures go up when unmarried cohabitations are included, although data sets on cohabitation status are notably difficult to obtain. A recent study [16] confirmed that non-marital cohabitations are overall less stable than marriages. They report that 49% of premarital cohabitations break up within 5 years (62% after 10 years), whereas 20% of marriages end up in separation or divorce within 5 years (33% after 10 years). A first stylized fact of the phenomenon we are looking at may thus be formulated as follows:

*Claim #*1: There is an epidemic failure in love relationships.

This notorious instability of sentimental relationships is not correlated with a significant loss of belief in the formulae of marriage or cohabitation as the main ingredient for happiness. On the contrary, people massively declare that a satisfactory sentimental relationship is the first element on which to build a happy life [1]. Moreover they also claim to want their partner to last them for life:

*Claim #*2: Couples typically conceive a relationship that lasts to be the main element in their pursuit of happiness. Moreover, most of them think that their own relationship will not collapse.

The available data supports claim #2. When asked to select the item that would make them happiest, 78% of college students in the US picked the one called: ‘falling and staying in love with your ideal mate’ [17]. In a national survey in the US [18], 93.9% of interviewed married couples thought their chances of a divorce or separation low (19.9%) or very low (74%), while 81.1% of unmarried respondents answered in the same way (32.4% low versus 47.7% very low).

It is intriguing that, in spite of the acknowledged high probability of breaking up, the vast majority of people think that their own relationship will not break down. Indeed, claims #1 and #2 together pose an apparent paradox. According to the data quoted above, a newly formed couple claims to be 90% certain that its own relationship will last. However the chances of breaking up after 5 years of cohabitation are 50%; and after 10 years it is definitely more probable than not that they will not be staying together. This fact could be stated as follows:

*The failure paradox:* how is it that a sentimental relationship planned to last will very probably break down?

The model proposed below shows that, under plausible assumptions, claims #1 and #2 are compatible. In order to test further the consistency of the model, we will consider two more stylized facts.

*Claim #*3: Couple disruption is the outcome of a gradual deterioration process.

The available data support this fact. According to 80% of all men and women interviewed in the California Divorce Mediation Project [19], the major reason given for their divorce was the ‘gradually growing apart and losing a sense of closeness, maybe staying together but emotionally detached until their loneliness is not longer bearable’.

*Claim #*4: The subjective well-being of partners decreases after marriage.

Although it is accepted that marriage goes with higher levels of happiness than singleness [1], [20], the average self-perceived satisfaction with life among those married is reported to peak around the time of marriage. This fact is supported by recent findings [21] –see also [22]. The pattern they find implies that, after marriage, the average reported satisfaction with life decreases (see figure number 2 in [21]).

### The Model

A simple dynamical model is formulated next that accounts for the scenario described above.

The core of the model lies in two key assumptions, namely the second law –to be discussed in A2 below– and the long-term planning of a couple's relationship –plausibly sustained by claim #2 above. These assumptions –along with weak homogamy (see A1 below) and a natural cost–benefit evaluation of the relationship state (assumption A3 below) –permit us to see the couple's sentimental relationship as an optimal control problem.

Modelling starts (time *t* = 0) when the romantic period is over and the feelings of partners about their relationship are at their peak (probably at the moment of commitment). At the initial time, the two partners, having an intense feeling for one another, agree on becoming a couple and undertake to do whatever is required to ensure a long future together. We assume:

**A1** (*Weak Homogamy*) Both partners share the same traits according to the model specifications below. Equivalently, the couple is the decision unit for the planning problem.

This assumption implies that the parameters, variables and utility structure defined in the model will all refer to the couple, as formed by two similar individuals. The fact that most people tend to feel attracted to individuals sharing the same traits they themselves posses has long been recognized in the literature [5], [11], [23], [24], [25]. Ample evidence in western societies supports this fact [12]. Thus assumption A1 stands as the rule, rather than the exception. In strict terms our theory only requires similarity in emotion rather than in personality between partners (see A3 below) although the two are shown to go together in dating and married couples [25].

As mentioned above, the following assumption is critical for our model.

**A2** (*Second law of thermodynamics for sentimental relationships*.) There is tendency for the initial feeling for one another to fade away. This kind of inertia must be counteracted by conscious practices.

There is general consensus in the literature about this fact [5], [9], [26], [27]. There seems to be a natural law that unattended love erodes as time goes by. Jacobson and Margolin [26] identified this fact as a major cause for marital instability. They write: ‘Marriages start off happy, but over time reinforcement erosion occurs that is the source of marital dysfunction’. The popular motto ‘love is not enough’ reflects this fact and implicitly suggests that erosion can be prevented somehow. The formulation of A2 as a law is taken from Gottman et al [9] (page 143), where the sentimental wearing out is suggestively explained as ‘something like a second law of thermodynamics for marital relationships: things fall apart unless energy is supplied to keep the relationship alive and well.’

In order to turn A2 into mathematics, a non-negative variable *x*(*t*) is defined to represent the state of the relationship at time *t*≥0. This is the *feeling variable* and it can be understood as the (common) sentiment that the partners have about one another. The variable *x*(*t*) serves as an ordinal variable probing the qualitative level of the relationship. Specific values of *x*(*t*) are uninformative, but the sentiment level at different times *t*_1_, *t*_2_ can be compared according to whether *x*(*t*_1_)≥*x*(*t*_2_) or *x*(*t*_1_)≤*x*(*t*_2_). At *t* = 0 the common feeling *x*(0) = *x*_0_ is assumed very large. We assume the relationship becomes unsatisfactory when *x*(*t*) falls below a certain threshold value *x*_min_ >0, which varies with the couple in question.

According to A2, the fading inertia can be counteracted by working on the relationship. This working will be represented by a non-negative and ordinal variable *c*(*t*) –called the *effort variable*– assumed piecewise continuous (see Appendix S1 about this). The scope of *c*(*t*) includes any everyday life practice serving as a reinforcement for the relationship. For instance, therapist suggest constructive actions (asking questions, listening actively, making plans together), and tolerant attitudes (accepting partners shortcomings, giving her/him privacy, respecting differences in tastes and habits), to name only a few among the recommended practices [5], [27]. The importance of effort/sacrifice, either passive or active, and its benefits on the relationship persistence have been widely recognized in the literature (see [28] for a review.)

A simple version of the second law can be written in terms of feeling and effort variables as the differential equation
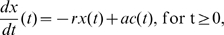
(1)with *r*>0 and *a*>0. Without intervention (i.e. *c*(*t*) = 0), Eq. (1) implies that *x*(*t*) fades at a constant rate *r*, specific to each relationship, which is a measure of the strength of feeling fading. This simple linear law is well-known to steer many natural and social phenomena. In fact, its discrete version was used in [9] to describe the baseline evolution of uninfluenced partner behaviour in short-term marital interaction. At any rate, Eq. (1) with *c*(*t*) = 0 is the first obvious working hypothesis for the decaying law of feeling. Effort enters as a recovery term in Eq. (1) counteracting the weakening of feeling. The parameter *a* obviously indicates *effort efficiency*. Selecting an effort plan *c*(*t*) determines the evolution of the feeling by solving Eq. (1) for *x*(*t*). Eq. (1) implicitly entails that *x*(*t*) changes smoothly, except at effort discontinuities.

The intensity of *c*(*t*) can be decided by the partners involved, in contrast to the level of the (non-rational) variable *x*(*t*), that cannot. The rational nature of the effort variable *c*(*t*) allows one to interpret it as a *control variable* in the scenario of optimal control theory [29]. In this setting, the controlled variable –the *state variable*– is *x*(*t*) and Eq. (1) is the *state equation* linking both variables.

Our next and last assumption refers to the cost-benefit valuation of effort and feeling levels. A standard utilitarian approach is considered. A mathematical representation of the emotional evaluation of feeling is rather straightforward (see A3 below). However formalization of effort valuation requires some considerations. The typical form of effort is sacrifice –forgetting one's self–interest for the sake of a close relationship–, whose potential benefits and costs have repeatedly been considered in the literature (see [30] and references therein.) Empirical research on sacrifice and related practices has evidenced that effort making may entail both emotional cost and benefits. This apparent contradiction is reconciled in [30] by means of a motivational analysis of sacrifice based on attitudes of approach and avoidance. While seeking to please one's partner wishes may lead to positive emotions, avoiding conflict may induce tension and distress. Our interpretation of the emotional differences in effort making is related to the intensity of effort since we consider effort to be emotionally rewarding up to a certain level but costly (distressing) beyond then. This is formalized as follows.

**A3** (*Utility structure*) There are two independent sources of utility. One comes from the level of feeling of attachment and the other is the consequence of the intensity of effort.

i) Utility from feeling is described by a differentiable function *U*(*x*) such that 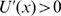, 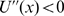, and 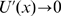 as *x*→∞. In words, for any feeling level, its marginal utility is positive and decreasing but it vanishes when feeling is large.
ii) Disutility of effort *c*≥0 is given by a differentiable function *D*(*c*) satisfying 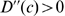, 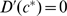 for some c*≥0, and 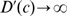 as *c*→∞. That is, effort dissatisfaction reaches its absolute minimum level at c* and marginal dissatisfaction goes up without bound as the effort level increases.

Notice that specific mathematical expressions for *U* and *D* are not required. The theory is valid for general functions as long as they satisfy the qualitative properties above.

The term utility may be interchanged with happiness, well-being or life satisfaction. The assumptions in part i) above are standard when utility depends on the consumption of some good. Utility defined on feeling is not an unnecessary superstructure: while *x* (how one feels) is directly linked to the (unprocessed) sentiment towards the relationship, *U*(*x*) produces a valuation of the feeling level *x* based on individual judgement and probably depends on past experiences or personality traits. For example, two different couples may attach quite different values to similar feeling levels, so that their valuations will be represented by different utility functions. The assumption on the existence of a utility function of feeling can be argued to be as sensible as it is in the case of utility dependent on consumption.

**Figure 1:**
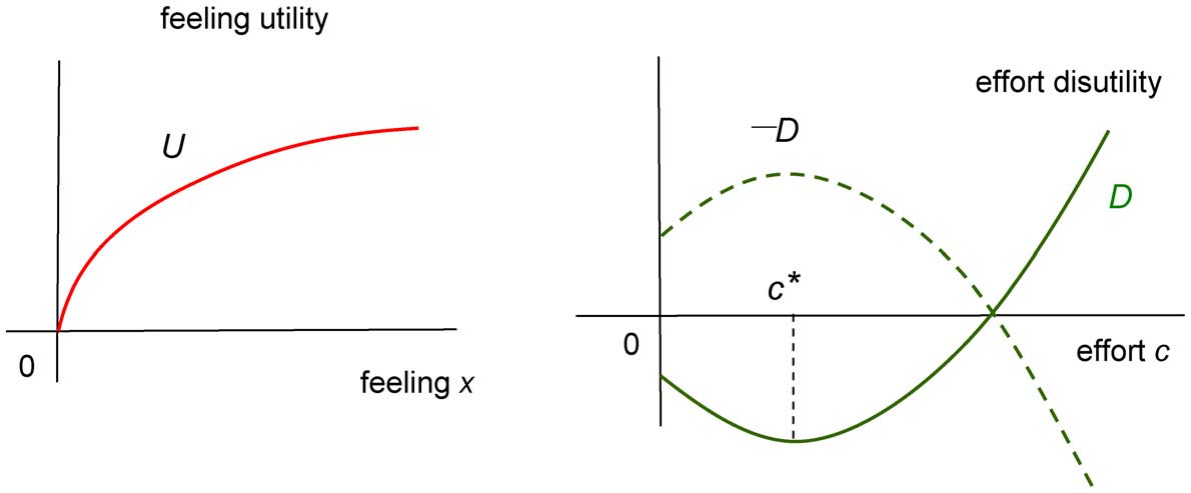
Utility structure: typical shapes of utility and disutility functions. The shape of feeling utility *U* is the standard picture assumed in the social sciences. Utility from effort, represented by −*D*, increases till it reaches c* but decreases beyond this point. Marginal effort utility is decreasing and vanishes at c*. Thus −*D* is concave in shape reaching an absolute maximum at c*.

The function *D* represents disutility, on the basis that making extra effort entails a cost in terms of utility. Its negative (−*D*) can thus be thought of as utility. The typical graphs of both functions are represented in Figure 1.

In the dynamic setting of the model, *U* and *D* mean to measure instantaneous utility and disutility, that is, of current levels of feeling and effort. The assumption that *D* may be non-monotonic leaves room for the fact that effort making may be felt as rewarding on its own within a certain range of low levels. To illustrate this, think of planning some recreational activity with your partner: it entails low effort and may certainly be enjoyable rather than distressing. Although future benefits of (current) effort making are implicitly taken into account via feeling utility –since current effort serves to enhance future feeling through equation (1)– the current benefits of effort making would not be admitted if *D* is always non-decreasing.

While making a small effort may plausibly be pleasant if the effort level is low, it is surely emotionally costly for sufficiently high effort levels. It is thus assumed in A3ii) above that making an additional effort increases utility until a level c* is reached, but decreases utility when the effort level goes beyond c*. The parameter c* thus corresponds to the *a priori* preferred effort level for the couple, and it plays a key role in the analysis. The theory admits *D* monotonic as a particular case, when c* = 0. This is the situation in which (current) effort generates (current) dissatisfaction from the very first effort unit. The proposed structure for *D* permits a more plausible situation.

The problem for a couple is how to design an effort policy that guarantees their relationship will endure and provide both partners with as much satisfaction as possible. The effort evolution is thus determined using an ideal criterion of pursuing maximal happiness. This is an optimality problem that can be formulated as follows.

**(P)** *The effort control problem for sentimental dynamics*: Assume feeling evolution given by Eq. (1), a utility structure as described in A3, initial feeling level *x*(0) = *x*_0_≫1, and denote the impatience factor by *ρ*>0. Under these conditions find the effort plan *c* ^♡^(*t*)≥0, for *t*≥0, that maximizes total discounted net utility and such that the associated evolution of both feeling and effort are sustainable in the long run.

Total satisfaction is obtained by aggregating discounted net instantaneous utilities for *t*≥0, which can be expressed –in a standard way– as
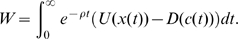
(the exponential term accounts for the discounted valuation of future utilities.) Problem **(P)** is a standard infinite horizon optimal control problem [29]. Because of claim #2, the planning period of the problem is considered unbounded. The issue of sustainability, a key requirement in the couple's problem, is concerned with two issues: admissibility and viability. Not only long term levels of both feeling and effort must be admissible (i.e. feeling must be kept above *x*_min_,), but also the transition to those asymptotic levels must be viable (see below.)

## Results and Discussion

The main implications of the model are derived and discussed next. Remarkably, the empirical evidence stated as claims #3 and #4 are derived theoretically from the model analysis. Also, claims #1 and #2 are shown to be compatible within the model framework, which somehow solves the failure paradox. The mathematical details of the analysis are placed in Appendix S1. The optimal (when positive) effort at time *t* must satisfy:
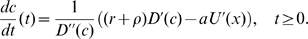
(2)Equation (2) gives the law of variation for optimal effort. Equations (1) and (2) form a system of differential equations for the optimal levels of feeling cum effort trajectories. These are denoted by (*x* ^♡^(*t*),*c* ^♡^(*t*)).

### Sentimental equilibrium

Stationary solutions of (1)–(2), if viable, guarantee a sustained happy sentimental life that is achieved on the basis of an invariant effort routine. Enjoying a permanent rewarding feeling, without turbulences in effort making, is obviously an attractive feature of a lasting sentimental dynamics. This makes equilibrium the desired configuration for a long term relationship.

#### Existence and viability

**Figure 2:**
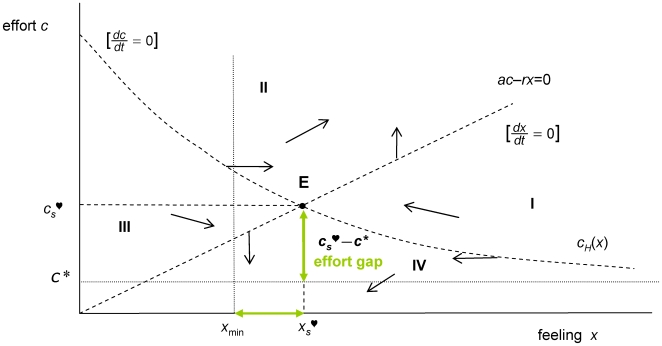
Sentimental equilibrium. Under the specifications of the model, there is always a unique feeling-effort equilibrium E of the optimal sentimental flow defined by Eqs. (1)–(2). This is a viable solution if *x*_s_^♡^>*x*_min_ and the effort gap *c*_s_^♡^−c* is tolerable. The vertical nullcline (where the sentimental flow is vertical, that is 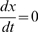) is the line *ac* = *rx* The horizontal nullcline is the curve *c_H_*(*x*) where the sentimental dynamics is flat, i.e. 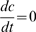, and its graph is always decreasing and located above the line *c* = c*. The graph represented above corresponds to the case that *U*′(0)<+∞, in turn implying *c_H_*(0)<+∞.

Equilibria are characterized by setting time derivatives equal to zero in (1)–(2). Under the specifications of the model, it is proved (Appendix S1) that there exists a unique well-defined sentimental equilibrium E = (*x*_s_^♡^,*c*_s_^♡^), which is depicted in Figure 2.

This is an admissible solution provided that *x*_s_^♡^ lies above *x*_min_. A crucial finding of the analysis is that the stationary effort level *c*_s_^♡^ lies above c* (see Appendix S1), as shown in Figure 2. This has the important implication that the extra effort 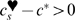 is needed to sustain the relationship dynamics in equilibrium. An equilibrium solution is viable provided the *effort gap* 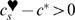 is not seen as too costly by the couple. A relationship is in equilibrium when 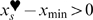 is admissible and the effort gap 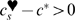 is comfortable. It will remain an equilibrium in the long run by fixing the constant effort plan *c*(*t*) = *c_s_* ^♡^. Since that level is the unique solution of **(P)** starting at E = (*x*_s_^♡^,*c*_s_^♡^), maximal well-being is achieved. However, the existence of the effort gap is a possible source of non-viability for the equilibrium solution.

#### Instability

A fundamental issue is whether or not perturbations will vanish or expand as time passes. If perturbations are amplified, the system is unstable. While stability contributes to a solid long life for the relationship, instability may be a serious drawback. In the unstable case, small shocks –typically due to lowering effort– will drive the feeling-effort configuration far from the equilibrium state. With no intervention, the final fate of the perturbed configurations is the dismantling of the relationship. This will be made clear in the analysis of the global sentimental dynamics. It is proved that the sentimental equilibrium defined by (1)–(2) is unstable (see Appendix S1). Furthermore, the local dynamics near equilibrium is of the saddle type. This has important implications for the global dynamics. A viable but unstable sentimental equilibrium is in principle sustainable, provided the couple is alert to correct perturbations that lower the stationary effort by injecting extra effort into the system and recover equilibrium.

### Sentimental kinetics and break-up mechanics

The initial state of the relationship is not generally placed at the equilibrium point because the initial feeling for each other is typically much higher than the stationary level *x*_s_^♡^. Therefore the discussion must proceed by looking at the dynamics (1)–(2) for an initial feeling *x*_0_≫*x*_s_^♡^. We need to look at the global configuration of the phase space to explain the transitory dynamics towards equilibrium.

#### Global sentimental dynamics

**Figure 3:**
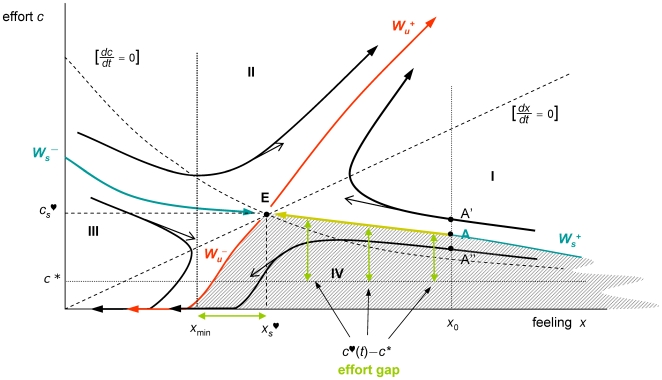
Durable relationships. Under the assumptions of the model, there is a unique effort policy that takes the initial feeling *x*_0_ to the unique equilibrium E. This is achieved by setting the initial effort at point A to get onto the stable manifold *W*_s_^+^ and then following path AE to approach equilibrium. Trajectories starting above *W*_s_^+^ (e.g. at point A′) are not acceptable. The target trajectory AE always lies above the line *c* = c*. The relationship is viable provided that the effort gap *c* ^♡^(*t*)−c* is tolerable along the transition to equilibrium, that must also satisfy *x*_s_^♡^>*x*_min_. Furthermore, since the target trajectory AE is unstable, trajectories starting at lower effort levels (e.g. at point A″) depart from AE and eventually lead to abandon effort (setting *c* = 0).

Figure 3 shows a qualitative picture of the feeling-effort phase space, obtained using standard techniques (see Appendix S1.) The picture is approximately valid for any utility and disutility functions satisfying assumption A3. The dynamical configuration is a nonlinear saddle. The oriented curves represent optimal trajectories (pieces of each trajectory maximize aggregate discounted net utilities for suitable initial and terminal conditions.)

The stable and unstable manifolds –composed of points (*x*,*c*) travelling to and from equilibrium– are represented by the curves *W*_s_ and *W_u_* in Figure 3 (each one split into two branches). Once a trajectory has reached the *x*-axis, effort must be optimally set at *c* ^♡^(*t*) = 0 from then onwards (see Appendix S1.) Thus, the continuation of a trajectory reaching the line *c* = 0 decays towards *x* = 0 along the *x*-axis, according to 
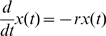
.

#### Transient dynamics for durable relationships

The key issue is whether or not, given an initial feeling *x*_0_, there exists an effort policy that leads to equilibrium and if it does what it is that characterizes the effort strategy. The stable manifold is the only curve supporting trajectories leading to equilibrium. Any other trajectory is either non acceptable or corresponds to a non-lasting relationship. Indeed, trajectories lying in regions I and II above *W*_s_ (see Figure 3) are not acceptable since they lead to increasingly higher levels of effort and in turn to unbearable disutility levels (see Appendix S1). On the other hand, trajectories reaching the *x*-axis in finite time lead to abandoning effort thereafter and eventually approach feeling levels that can only lead to the end the relationship. Given *x*_0_, there is a suitable level *c*_0_^♡^ for which A = (*x*_0_,*c*_0_^♡^) lies in *W*_s_^+^ and evolves towards E (Figure 3.) This *target trajectory* AE represents the (unique) recipe for a lasting successful relationship, provided *x*_s_^♡^ is greater than *x*_min_. Since the target trajectory AE embedded in *W*_s_^+^ lies entirely above the line *c* = c*, an amount of extra effort (greater than c*) must be made along the path to equilibrium. Therefore, two conditions are required for an optimal trajectory AE to be successful, namely, the feeling surplus 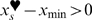 must be rewarding and the effort gap 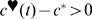 must be tolerable for *t*≥0. The presence of the effort gap along the target path may be a source of couple disruption, since it can possibly be tolerated with difficulty in many cases.

#### Decreasing well-being

Since the target path lies in region I, *c* ^♡^(*t*) increases while *x* ^♡^(*t*) decreases in the path to equilibrium values. It follows from A3 and the chain rule that 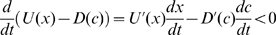. This means that well-being decreases along the optimal path AE until reaching E. This theoretical prediction of the model is in accordance with claim #4, stated in section 2.

#### Break-up mechanics

As explained above, typical dynamics occur within the shaded region in Figure 3, for *x*_0_ is large and trajectories leading to increasing levels of effort are not plausible. Along trajectories in the shaded area, the effort eventually decreases until the *x*-axis is hit and it then optimally settles at *c* ^♡^(*t*) = 0. This makes the relationship no longer viable in the medium/long run. Because of instability a deviation, induced by a reduction in effort, from a trajectory initially settled at *W*_s_^+^ leads the state of the system into the shaded region, where optimal trajectories diverge from the target curve. This critical feature is the main source of sentimental instability.

**Figure 4:**
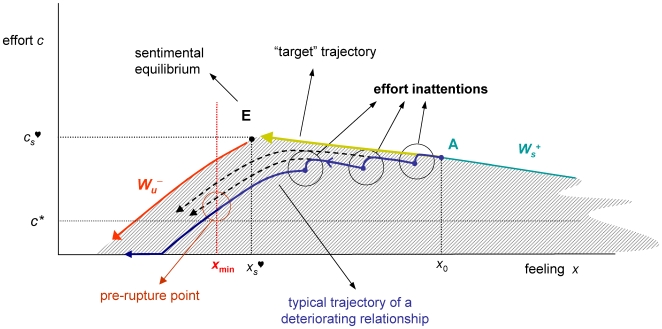
Breakup mechanics. The model produces a plausible scenario, through a sequence of effort inattentions, for the deterioration of a relationship in a gradual form, which seems to be typical according to data. Because of the effort gap, there is a tendency to lower the right effort level. Then the intrinsic instability of sentimental dynamics obeying the second law causes the piecewise decaying trajectories to move further and further away from the target trajectory and eventually to cross the threshold level *x*_min_. This is considered a point of *pre-rupture*, since it is a matter of time before effort is abandoned.

A possible mechanism –via *effort inattentions*– accounting for the gradual deterioration of a relationship is displayed in Figure 4 and can be described as follows. Assume that the relationship initially configured at state A follows for some time the target trajectory AE on the stable branch *W*_s_^+^. If at a certain point effort inattention occurs, that is if the effort level is lowered, the state is driven out of *W*_s_^+^. If effort is not returned to the correct level and if the system follows the optimal dynamics (1)–(2), the new deviated state finds itself at an initial condition of a trajectory moving away from the target trajectory. This new trajectory may be followed for a while until new effort inattention occurs, expelling the state to a new position with lower effort level, in turn following a new decaying trajectory moving further away from *W*_s_^+^. Through a sequence of effort inattentions, instability causes the decaying trajectories to cross the threshold level *x*_min_ (Figure 4.) This is a point of *pre-rupture*, since feeling falls below satisfactory levels and it is a matter of time before effort is abandoned. The relationship might go on for the time being but eventually will reach unendurable conditions. This final stage in which emotional attachment gradually disappears matches the description of the majority of divorces described in [19].

If the system is following a decaying trajectory, the target path dynamics can be restored by increasing the effort level. However, the longer it takes to react and correct deviations, the farther the state is from the target path, and the more difficult it is to restore the system to the lasting path. If effort is neglected for too long, it may become irreversible. A considerable amount of reported unhappy marriages seem to fit this diagnosis [3], [9]. The deteriorating process described above is consistent with claim #3 in section 2.

### Closing remarks

The mathematical theory introduced in this paper unveils an underlying mechanism that may explain the deterioration and disruption occurring massively in sentimental relationships that were initially planned to last forever. Two forces work together to ease the appearance of the deterioration process. First, it happens that since an extra effort must always be put in to sustain a relationship on the successful path, partners may relax and lower the effort level if the gap is uncomfortable. Then instability enters the scene, driving the feeling-effort state out of the lasting successful dynamics.

A further significant finding is the fact that partners construct and perceive their relationships as definitive projects is compatible with the evidence that their union may probably fall apart –which is typical in the model dynamics. This dismantles the failure paradox, accounting for probable couple disruption as a gravitational consequence of the second law under optimality.

The model analysis may offer advice to partners about how to keep a long term relationship afloat. Lasting relationships are possible only if the effort gap is tolerable and the optimal effort making is continuously watched over to stay on the target dynamics. A realistic lasting relationship, when the effort gap is satisfactory, may be described by a trajectory travelling near the stable branch for a while and then wandering near equilibrium alert at keeping effort at the right level. These kinds of relationships are seen often enough although they may appear exceptional. This is consistent with the exceptionality of durable successful relationships within the model.

Two apparent facts serve as a first test to validate the theory proposed in this paper: (i) the model formulation builds on accepted evidence (namely, the second law and the intention of couples to design their relationships to last forever) and (ii) the mathematics of the model shows consistency with further empirical facts on divorce and separation, namely the typical progressive deterioration of failing relationships (which is claim #3 in section 2) and the decrease of well-being after marriage (claim #4 in section 2). Further research to validate the model should address testing –in a lab experiment or a field survey– the two main findings of the theory, i.e. the existence of the effort gap and the unstable nature of feeling-effort dynamics.

The pessimistic conclusions for couple durability should remain valid in a less ideal scenario as long as the formulation of the second law is considered valid. More realistic assumptions like (weak) heterogamy, presence of external shocks or sub-optimal behaviour, probably enter the scene as contributing factors enforcing instability. The effort gap plus the unveiled instability identify an essential intrinsic mechanism for probable sentimental failure.

## Supporting Information

Appendix S1 Supporting document containing the mathematical derivations for the analysis in the main manuscript.
(0.16 MB DOC)
