# Supplementary material for: A Mathematical Model of Sentimental Dynamics Accounting for Marital Dissolution
Source: PLoS One. 2010 Mar 31;5(3):e9881. doi: 10.1371/journal.pone.0009881 (PMC2847599; doi:10.1371/journal.pone.0009881)
Supplement: Appendix S1 — Supporting document containing the mathematical derivations for the analysis in the main manuscript. (0.16 MB DOC) [file pone.0009881.s001.doc]

**Appendix**

The mathematical derivations below follow the line of argument developed in section 3. The basic facts of optimal control theory can be found in many textbooks, e.g. [29].

In order to apply optimal control theory, technical assumptions on the differentiability of state and control variables are required [29]. Specifically, the state variable (the feeling in the model) is assumed to be continuous with piecewise continuous derivative so, not only *x*(*t*) is continuous but its variation also is except for a finite number of points. Also, the control variable (the effort) is assumed piecewise continuous. These assumptions seem to fit well within the framework of the sentimental model for, while gaps in the effort may be reasonably expected to occur at some points –so thus *c*(*t*) will have a jump discontinuity at such points–, the feeling is plausibly expected to vary smoothly, except at a point of effort gap.

**Derivation of the optimal feeling-effort system.** The current-value Hamiltonian for the control problem is given by $H(x,c,m)=U(x)-D(c)+m(-rx+ac)$, where *m*(*t*) is the current-value co-state variable. Since *H* is strictly concave with respect to *c*, a sufficient condition for an interior optimal control is $-D'(c)+am=0$. Taking time derivatives in this expression we have $\frac{dm}{dt}=\frac{1}{a}D''(c)\frac{dc}{dt}$. The co-state equation is $\frac{dm}{dt}=-U'(x)+(r+\rho)m$. Combining the last three equations, we obtain

$\frac{dc}{dt}=\frac{1}{D''(c)}\left((r+\rho)D'(c)-aU'(x)\right)$,

which is Eq. (2). The case of a corner maximum of *H* is considered below.

**Location of nullclines and existence of a unique equilibrium.** Equilibria are located at the intersection of the nullclines, i.e. loci of the plane (*x*,*c*) where movement is parallel to the axes. They are defined by $ac=rx$ (vertical nullcline), and $(r+\rho)D'(c)=aU'(x)$ (horizontal nullcline). The horizontal nullcline can be described as a function $c=c_{H}(x)$. Differentiating with respect to *x* we get:

$(r+\rho)D''(c)c'_{H}(x)=aU''(x)$,

Therefore $c'_{H}(x)<0$ in view of A3. Since $D''(c)>0$, by A3 we have

$D'(c_{H}(x))=\frac{aU'(x)}{r+\rho}>0=D'(c*)$,

so that $c_{H}(x)>c*$, i.e. the horizontal nullcline is above the line *c*=*c**. Since $D'(c_{H}(x))\to0$ as $x\to\infty$, $c_{H}(x)$ asymptotically approaches the line *c*=*c** as $x\to\infty$*.* If $U'(x)\to+\infty$ as $x\to0$, then *c_H_*(*x*) approaches infinity as $x\to0$*.* In the case that $U'(0)<+\infty$*,* the graph of *c_H_*(*x*) is shown in Figure 2. In any case the nullclines intersect at a point E(*x*_s_^^,*c*_s_^^)*,* which corresponds to the unique equilibrium of the system.

**Instability of equilibrium.** Since the determinant of the Jacobian *J*_E_ of the system (1)−(2) at E is negative, by A3:

$\det J_{\text{E}}=-r(r+\rho)+a^{2}\frac{U''(x_{s}^{♥})}{D''(c_{s}^{♥})}<0$,

the equilibrium is a saddle and therefore unstable.

**Global dynamics.** The two nullclines −represented by the dashed lines in Figure 2− divide the phase space into four regions, denoted by I, II, III and IV, in which the local direction of displacement of the trajectories (*x*^^(*t*),*c*^^(*t*)) points northwest, northeast, southeast, and southwest, respectively. This information suffices to locate approximately the stable and unstable manifolds of the saddle-type dynamics. The consistency of a saddle dynamics implies that the stable branch *W_s_^+^* must lie entirely in region I. The global picture of the phase space is displayed in Figure 3. Notice that the picture is valid qualitatively for any utility and disutility functions satisfying assumption A3.

**Continuation of trajectories hitting the *x*-axis.** The case in which the optimal effort is null must be treated separately, since Eq. (2) need not be satisfied in that situation. Since *H* is strictly concave, the optimal effort at time *t* is *c*(*t*)*=*0 if and only if $m(t)\leq\frac{1}{a}D'(0)$. It thus holds that for *m*(*t*) and *c*(*t*)≥0 optimal. Then, for any trajectory lying in region IV,$m(t)\leq\frac{1}{a}D'(c(t))<\frac{1}{r+\rho}U'(x(t))$, and thus $\frac{dm}{dt}=-U'(x)+(r+\rho)m<0$ for *t*0. It follows from the global dynamics that any trajectory lying below the stable manifold eventually ends hitting the *x*-axis in finite time, so that *c*(*t*_0_)*=*0 and for some *t*_0_. Since *m*(*t*) is decreasing in region IV, $m(t)\leq\frac{1}{a}D'(0)$ for *t**t*_0_ and in turn *c*(*t*)=0 for *t**t*_0_ optimally. Therefore, the optimal continuation of a trajectory intercepting the *x*-axis at some point in time, is obtained by setting effort to zero from then on.

**Unbearable levels of disutility along trajectories lying above the stable manifold.** Trajectories lying above *W_s_* eventually enter region II (Figure 4), where $\frac{dx}{dt}>0$ and $\frac{dc}{dt}>0$. It also holds that $\frac{dc}{dx}>0$ which cannot approach zero as $t\to\infty$. Thus, for these trajectories A3 implies that, as $t\to\infty$,

$\frac{d}{dt}\left(U(x(t))-D(c(t))\right)=\frac{dx}{dt}\left(U'(x(t))-D'(c(t))\frac{dc}{dx}\right)\xrightarrow{}-\infty$,

since the global dynamics prevents $\frac{dx}{dt}$ from approaching zero as $t\to\infty$. This implies that (current) net utility decreases without limit as times increases.
